# Association of Tumor Stage and Molecular Subtype With Three-Year Recurrence Outcomes After Adjuvant Radiotherapy in Stage I-III Breast Cancer: A Single-Centre 2022 Cohort Study

**DOI:** 10.7759/cureus.108710

**Published:** 2026-05-12

**Authors:** Nusrat Nazar, Ahmad R Ghumman, Abdul S Waqar, Mohammad A Sahi, Aleesha Ilyas, Naeem Haider

**Affiliations:** 1 Oncology, Cancer Care Hospital & Research Centre, Lahore, PAK

**Keywords:** adjuvant radiotherapy, breast cancer, distant metastasis-free survival, locoregional recurrence-free survival, molecular subtype, recurrence-free survival

## Abstract

Background

Breast cancer remains a major clinical challenge, and recurrence after curative-intent treatment continues to influence prognosis and follow-up strategies. Tumor stage and molecular subtype are central to treatment planning and risk stratification, yet real-world recurrence data from low- and middle-income country (LMIC) settings, where treatment access, timing, and completion may diverge substantially from controlled trial environments, remain limited. We evaluated the association of stage and molecular subtype with three-year recurrence outcomes in a single-center Stage I-III breast cancer cohort treated with adjuvant radiotherapy.

Methods

This retrospective cohort study included consecutive Stage I-III breast cancer patients diagnosed in 2022 and treated with adjuvant radiotherapy at a tertiary cancer centre in Lahore, Pakistan. The primary endpoint was recurrence-free survival (RFS), defined from the biopsy date to first locoregional recurrence, distant recurrence, or death. Secondary endpoints were distant metastasis-free survival (DMFS) and locoregional recurrence-free survival (LRRFS). Kaplan-Meier curves with log-rank testing compared outcomes by stage group (I-II vs III) and molecular subtype. Multivariable Cox proportional hazards regression was used for adjusted RFS and DMFS analyses, with a further radiotherapy-start-anchored sensitivity analysis for RFS.

Results

Of 195 patients analyzed, 115 (59.0%) had Stage I-II disease, and 80 (41.0%) had Stage III disease. Molecular subtypes were Luminal A (57.4%), Luminal B (17.9%), HER2-enriched (10.8%), and triple negative (13.8%). RFS events occurred in 64 patients: 54 unique patients experienced recurrence, including seven with both distant and locoregional recurrence, while 10 additional patients died without prior documented recurrence. Stage III disease was independently associated with significantly worse RFS (hazard ratio (HR) 2.182, 95% CI 1.266-3.761; p = 0.005) and DMFS (HR 2.429, 95% CI 1.337-4.411; p = 0.004) in multivariable models. The stage effect persisted in both sensitivity analyses. Molecular subtype was not significantly associated with any recurrence endpoint on univariable or multivariable testing.

Conclusion

Tumor stage was the most consistent and robust predictor of recurrence outcomes after adjuvant radiotherapy in this single-centre cohort. Although molecular subtype did not independently predict recurrence in these analyses, likely reflecting statistical limitations inherent to smaller, real-world datasets, these findings support stage-based risk stratification in routine clinical practice. Larger multicentre cohorts with complete systemic treatment data are needed to better characterise subtype-specific recurrence patterns in LMIC settings.

## Introduction

Breast cancer is the most frequently diagnosed cancer in women across 157 of 185 countries worldwide. According to the World Health Organization, approximately 2.3 million women were diagnosed with breast cancer, and nearly 670,000 died from the disease in 2022, making it a leading global health burden [1]. While incidence rates are highest in high-income countries, mortality rates are disproportionately elevated in low- and middle-income settings due to late-stage presentation, limited diagnostic infrastructure, and gaps in treatment access [2].

Adjuvant radiotherapy is an established component of curative-intent treatment for early and locally advanced breast cancer. Following breast-conserving surgery, radiotherapy reduces the risk of locoregional recurrence by roughly 60%-70%, and post-mastectomy radiotherapy further decreases both locoregional and systemic failure in selected high-risk patients [3-4]. Nonetheless, real-world recurrence outcomes after adjuvant radiotherapy vary considerably across institutions and settings because of differences in patient mix, disease stage at presentation, referral patterns, treatment sequencing, and completion. Data from low- and middle-income country (LMIC) cancer centres, in particular, are under-represented in the published literature, limiting the applicability of trial-derived estimates to these populations.

In contemporary breast cancer management, stage and molecular subtype, broadly defined by hormone receptor and HER2 status as Luminal A, Luminal B, HER2-enriched, or triple negative, are the two cornerstones of prognostication and treatment planning [5]. Molecular subtype is a recognised prognostic marker associated with differences in recurrence timing, pattern, and response to systemic treatment across large trial and registry cohorts [6]. However, the magnitude of subtype-specific associations with recurrence endpoints in single-centre observational datasets may be attenuated by limited subgroup sizes, heterogeneity in adjuvant systemic therapy delivery, and incomplete treatment-completion data. In our institution, detailed completion data for trastuzumab and endocrine therapy were not consistently available for this 2022 cohort, making a descriptive association-focused analysis the most methodologically appropriate approach.

This study aimed to evaluate the association of tumour stage and molecular subtype with three-year recurrence outcomes in Stage I-III breast cancer patients treated with adjuvant radiotherapy at a single tertiary cancer centre in Pakistan. We prespecified recurrence-free survival (RFS) as the primary endpoint and distant metastasis-free survival (DMFS) and locoregional recurrence-free survival (LRRFS) as secondary endpoints. An additional sensitivity analysis used the radiotherapy start date as the time origin to assess the robustness of the primary findings.

## Materials and methods

Study design and setting

This retrospective single-centre cohort study was conducted at Cancer Care Hospital & Research Centre, Lahore, Pakistan. The cohort comprised patients with Stage I-III breast cancer diagnosed in 2022 who received adjuvant radiotherapy as part of curative-intent treatment. The study was prepared and reported in accordance with the Strengthening the Reporting of Observational Studies in Epidemiology (STROBE) statement for cohort studies [7].

Participants

Patients were eligible for inclusion if they had histologically confirmed Stage I-III breast cancer, underwent definitive surgery (breast-conserving surgery or mastectomy), and received adjuvant radiotherapy with available baseline clinicopathologic characteristics and date variables required for survival analysis. Patients with metastatic disease at presentation (Stage IV), missing key time variables that precluded endpoint construction, or non-adjuvant radiotherapy intent were excluded. After applying these criteria, the final analyzed cohort consisted of 195 patients.

Variables and data sources

Data were abstracted from institutional clinical and pathology records. Variables collected included age at diagnosis, stage grouping (Stage I-II vs III), molecular subtype (Luminal A, Luminal B, HER2-enriched, triple negative), surgery type (breast-conserving surgery vs mastectomy), chemotherapy category, surgical margin status, radiotherapy type and modality, total radiotherapy dose (Gy), and number of fractions. Date variables included biopsy date, surgery date, radiotherapy start and end dates, last follow-up date, death date, and dates of distant and locoregional recurrence. For patients receiving neoadjuvant chemotherapy (NACT), pretreatment clinical stage was used for stage grouping, rather than post-treatment pathological downstaging, to preserve baseline risk stratification.

Molecular subtype was assigned using ER, PR, HER2, and Ki-67 status. Ki-67 was recorded as a binary institutional pathology category, low or high, rather than as a continuous percentage value. Luminal A was defined as hormone receptor-positive, HER2-negative disease with low Ki-67. Luminal B included hormone receptor-positive tumours with HER2 positivity and/or high Ki-67. HER2-enriched disease was defined as ER/PR-negative and HER2-positive, while triple-negative disease was defined as ER/PR/HER2-negative.

For multivariable analyses, stage was grouped as Stage I-II versus Stage III. Age was modelled as a continuous variable per 10-year increment (age10). For the treated-only sensitivity model, chemotherapy was recoded as NACT vs adjuvant; patients with no chemotherapy (n = 2) were coded as missing for this binary variable and excluded from that model.

Outcome definitions

The primary endpoint, RFS, was defined from the biopsy date to the first occurrence of locoregional recurrence, distant recurrence, or death from any cause, whichever occurred first. Patients without an RFS event were censored at the last known contact date.

Secondary endpoints were DMFS, defined from biopsy date to distant recurrence (death without prior recurrence treated as censoring), and LRRFS, defined from biopsy date to locoregional recurrence (death without prior recurrence treated as censoring). 

For RFS, each patient was counted once according to the first event contributing to RFS. Distant recurrence, locoregional recurrence, and death were also tabulated separately as follow-up outcomes; therefore, these categories were not mutually exclusive. Patients who experienced both distant and locoregional recurrence were identified as an overlap group, and deaths after a previous recurrence were not counted as additional RFS events.

A prespecified sensitivity analysis for the primary RFS endpoint used radiotherapy start date as the time origin (RT-start anchored RFS), with the same event definition otherwise preserved.

Data preparation and quality checks

Date variables were converted to standardised date fields and validated using prespecified sequence checks, including confirmation that no radiotherapy end date preceded its corresponding start date, that surgery dates were not recorded before biopsy dates, and that recurrence dates were not logged before radiotherapy initiation. All sequence-flag violations identified during data preparation were resolved prior to final analysis, and the cleaned dataset contained no remaining flag violations.

Statistical analysis

Continuous variables are summarised as mean ± standard deviation (SD) or median and range as appropriate; categorical variables are summarised as counts and proportions. Kaplan-Meier curves were generated for RFS, DMFS, and LRRFS stratified by stage group and molecular subtype. Between-group differences were assessed with the log-rank (Mantel-Cox) test. Median survival estimates were not reached for several subgroup analyses because of limited follow-up duration and censoring patterns; therefore, estimated mean event-free survival times are reported. Three-year event-free estimates were taken from Kaplan-Meier survival tables at 36 months or the closest preceding available time point.

Multivariable Cox proportional hazards regression was performed for the primary RFS endpoint (main model covariates: age10, stage group, molecular subtype, surgery type) and for a treated-only sensitivity RFS model that additionally included the NACT vs adjuvant chemotherapy binary. A further RT-start-anchored Cox sensitivity model was run for RFS. For DMFS, a parsimonious Cox model (age10, stage group, subtype) was used in view of the lower event count. Multivariable Cox regression was not performed for LRRFS because only 13 events occurred, creating an unacceptable risk of model instability. Hazard ratios (HRs) with 95% confidence intervals (CIs) and two-sided p-values are reported; p < 0.05 was considered statistically significant. Kaplan-Meier and Cox regression analyses were conducted in IBM SPSS Statistics Version 29.0 (IBM Corp., Armonk, NY, USA). Competing-risk analyses were conducted in R using Fine-Gray regression methods with the cmprsk package (R Foundation for Statistical Computing, Vienna, Austria). The proportional hazards assumption was assessed using Schoenfeld residuals.

As a sensitivity analysis for recurrence-specific secondary endpoints, competing-risk analyses were performed for DMFS and LRRFS, treating death without prior endpoint-specific recurrence as a competing event. Cumulative incidence functions and Fine-Gray subdistribution hazard models were used to estimate subdistribution HRs (sHRs) with 95% CI. For DMFS, the Fine-Gray model included age per 10 years, stage group, and molecular subtype. For LRRFS, only a stage-based Fine-Gray model was performed because the number of locoregional recurrence events was low.

Ethical considerations

This study was conducted in accordance with the Declaration of Helsinki and local institutional ethical requirements. Ethics approval was obtained from the Institutional Review Board (IRB) of Cancer Care Hospital & Research Centre (approval number: IRB/CCH&RC/0708/25). Given the retrospective design and use of routinely collected clinical data, a waiver of informed consent was applied as per institutional approval.

## Results

Cohort characteristics

The final analysed cohort comprised 195 patients. Stage I-II disease was present in 115 patients (59.0%) and Stage III disease in 80 (41.0%). The most common molecular subtype was Luminal A (n = 112, 57.4%), followed by Luminal B (n = 35, 17.9%), triple negative (n = 27, 13.8%), and HER2-enriched (n = 21, 10.8%). The majority of patients underwent mastectomy (n = 119, 61.0%) rather than breast-conserving surgery (n = 76, 39.0%), and nearly all patients received hypofractionated radiotherapy (n = 194, 99.5%). The mean age at diagnosis was 49.45 ± 10.39 years. Mean radiotherapy fractions and dose were 16.05 ± 1.97 Gy and 43.13 ± 5.59 Gy, respectively. Radiotherapy dose and fractionation data were missing for two patients; both completed treatment and were included in all time-to-event analyses. Radiotherapy parameters are, therefore, reported for 193 patients. Full baseline characteristics are presented in Table 1.

**Table 1:** Baseline clinicopathologic and treatment characteristics of the cohort (n = 195). RT: radiotherapy; RFS: recurrence-free survival; SD: standard deviation

Variable	Category / Summary	Value, n (%) / Mean ± SD
Age at diagnosis	Mean ± SD (n = 195)	49.45 ± 10.39 years
Stage group	Stage I–II	115 (59.0%)
	Stage III	80 (41.0%)
Molecular subtype	Luminal A	112 (57.4%)
	Luminal B	35 (17.9%)
	HER2-enriched	21 (10.8%)
	Triple negative	27 (13.8%)
Ki-67	Low	141 (72.3%)
	High	54 (27.7%)
Surgery type	Breast-conserving surgery	76 (39.0%)
	Mastectomy	119 (61.0%)
Chemotherapy category	Neoadjuvant chemotherapy	104 (53.3%)
	Adjuvant chemotherapy	89 (45.6%)
	No chemotherapy	2 (1.0%)
Margins	Clear	190 (97.4%)
	Involved	4 (2.1%)
	Not assessed	1 (0.5%)
Radiotherapy type	Hypofractionated	194 (99.5%)
	Conventional	1 (0.5%)
RT fractions	Mean ± SD (n = 193)	16.05 ± 1.97
RT dose	Mean ± SD (n = 193)	43.13 ± 5.59 Gy
Outcome events	RFS events	64 (32.8%)
	Distant recurrence	48 (24.6%)
	Locoregional recurrence	13 (6.7%)
	Deaths	33 (16.9%)

During follow-up, 48 patients developed distant recurrence, and 13 developed locoregional recurrence; these categories were not mutually exclusive. Seven patients had both distant and locoregional recurrence. Therefore, 54 unique patients experienced at least one documented recurrence: 41 distant recurrence only, six locoregional recurrence only, and seven both distant and locoregional recurrence. The 64 RFS events comprised these 54 unique recurrence patients plus 10 deaths without previous documented recurrence. Of the 33 deaths observed during follow-up, 23 occurred after or with documented recurrence and were therefore not counted as additional RFS events. Median follow-up duration was 40.9 months using the reverse Kaplan-Meier method. Overall, 162 of 195 patients (83.1%) had at least 36 months of potential follow-up from biopsy date to last contact or death.

Kaplan-Meier analyses

**Figure 1:**
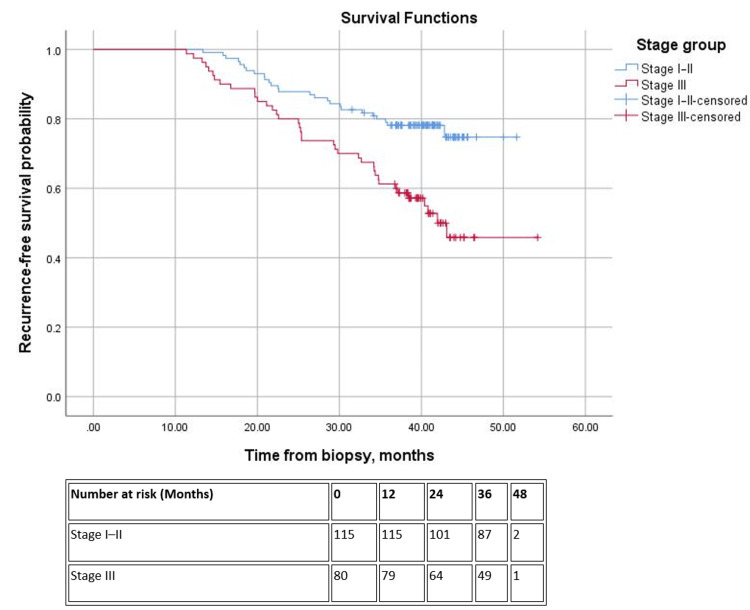
Kaplan–Meier recurrence-free survival (RFS) curves stratified by stage group (Stage I–II vs Stage III). Numbers at risk are shown at 0, 12, 24, 36, and 48 months. The figure is generated using IBM SPSS Statistics for Windows, Version 29.0 (IBM Corp., Armonk, NY, USA).

Stage significantly differentiated RFS. Among 115 Stage I-II patients, 26 experienced an RFS event; among 80 Stage III patients, 38 did so (log-rank chi-square = 12.308, df = 1, p < 0.001). The estimated three-year RFS was approximately 78.1% for Stage I-II versus 60.0% for Stage III. Mean RFS was 45.39 months (95% CI 43.22-47.57) in Stage I-II and 40.17 months (95% CI 36.76-43.58) in Stage III. The log-rank test demonstrated significantly worse RFS in Stage III disease (p < 0.001). Numbers at risk are indicated below the x-axis at each time interval in Figure 1.

**Figure 2:**
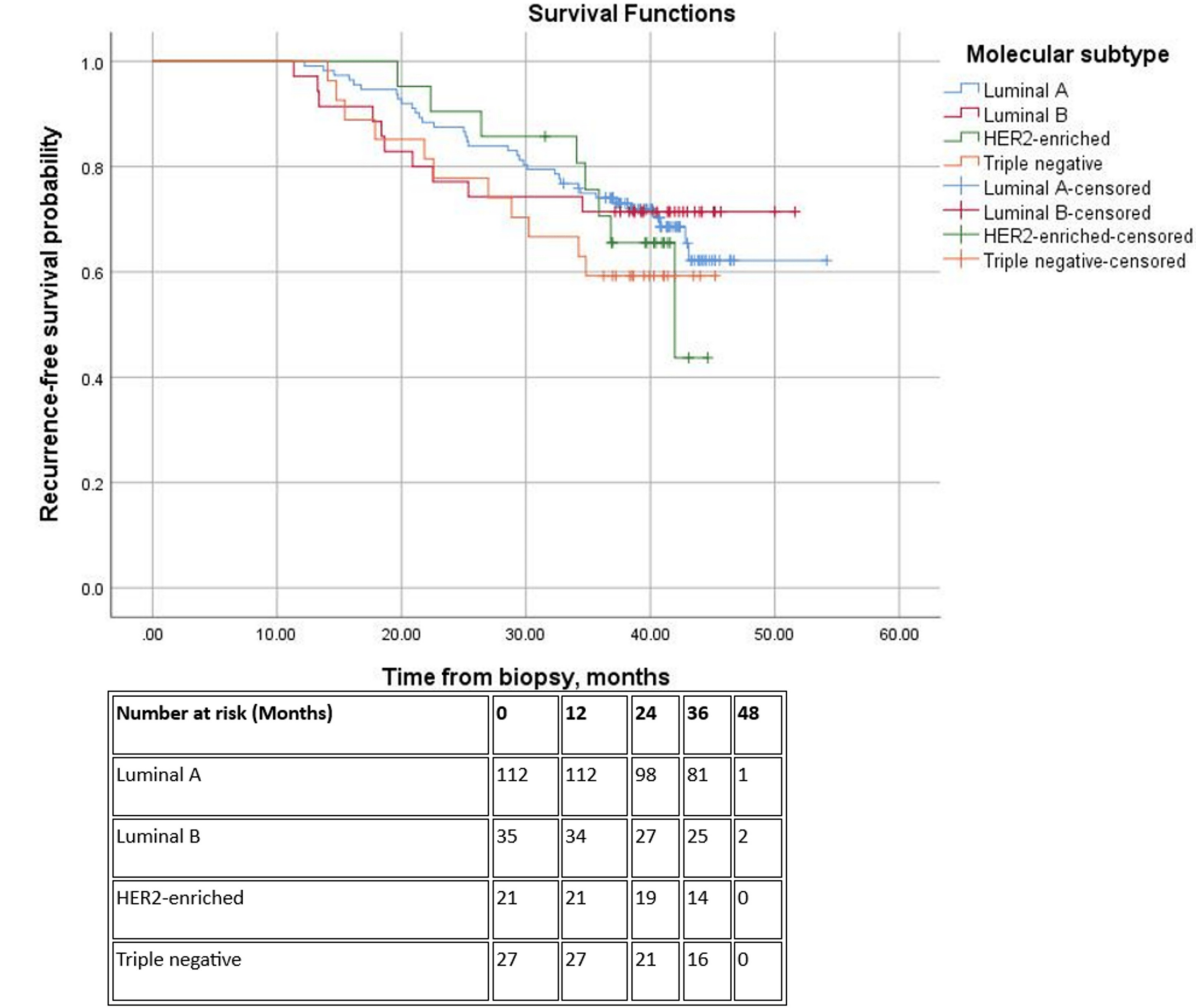
Kaplan–Meier RFS curves stratified by molecular subtype (Luminal A, Luminal B, HER2-enriched, triple negative). Numbers at risk are shown at 0, 12, 24, 36, and 48 months. The figure is generated using IBM SPSS Statistics for Windows, Version 29.0 (IBM Corp., Armonk, NY, USA).

No statistically significant difference in RFS was observed across the four molecular subtypes on global log-rank testing (chi-square = 1.573, df = 3, p = 0.666). Event counts were 35 of 112 for Luminal A, 10 of 35 for Luminal B, 8 of 21 for HER2-enriched, and 11 of 27 for triple-negative disease. Although the mean RFS was numerically shorter in the HER2-enriched and triple-negative subgroups, CIs overlapped substantially, and global log-rank testing did not show a statistically significant difference (p = 0.666) (Figure 2).

**Figure 3:**
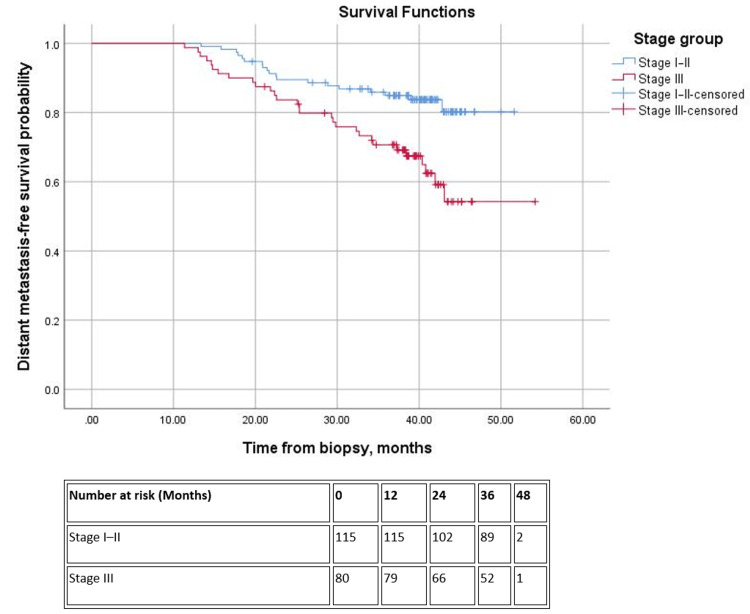
Kaplan–Meier distant metastasis-free survival (DMFS) curves stratified by stage group. Numbers at risk are shown at 0, 12, 24, 36, and 48 months. The figure is generated using IBM SPSS Statistics for Windows, Version 29.0 (IBM Corp., Armonk, NY, USA).

DMFS also differed significantly by stage. Distant recurrence occurred in 19 of 115 Stage I-II patients and 29 of 80 Stage III patients (log-rank chi-square = 9.753, df = 1, p = 0.002). The estimated three-year DMFS was approximately 85.0% for Stage I-II and 70.7% for Stage III. Stage III disease was associated with significantly worse DMFS (p = 0.002) (Figure 3).

**Figure 4:**
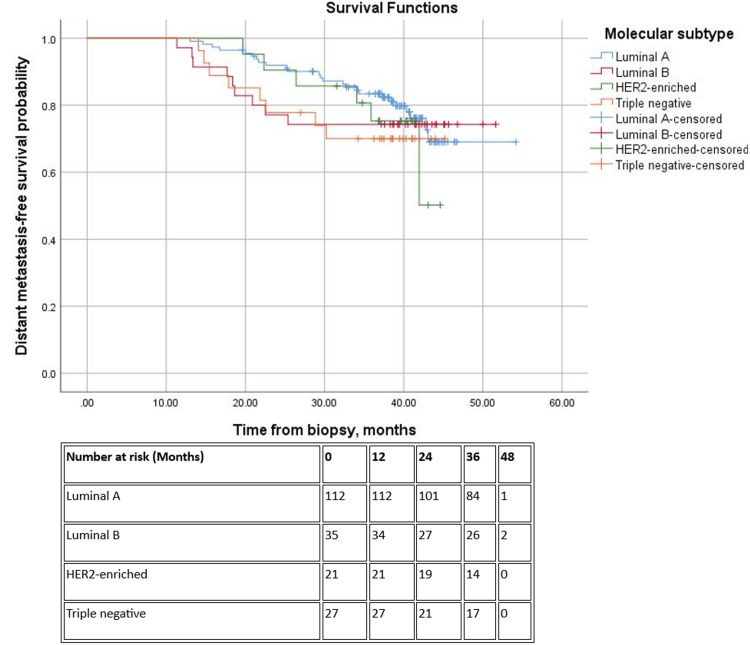
Kaplan–Meier distant metastasis-free survival (DMFS) curves stratified by molecular subtype (Luminal A, Luminal B, HER2-enriched, triple negative). Numbers at risk are shown at 0, 12, 24, 36, and 48 months. The figure is generated using IBM SPSS Statistics for Windows, Version 29.0 (IBM Corp., Armonk, NY, USA).

No significant difference in DMFS was observed by molecular subtype on global log-rank testing (p = 0.722) (Figure 4).

**Figure 5:**
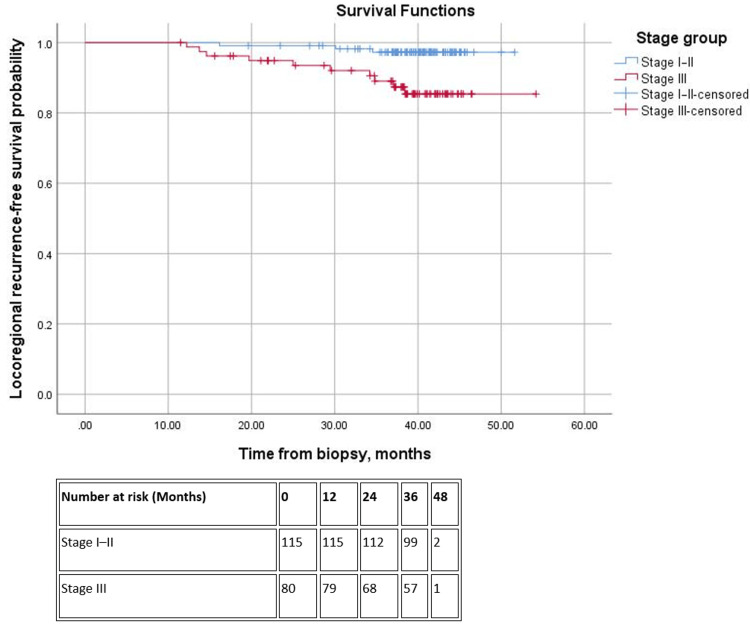
Kaplan–Meier locoregional recurrence-free survival (LRRFS) curves stratified by stage group. Numbers at risk are shown at 0, 12, 24, 36, and 48 months. The figure is generated using IBM SPSS Statistics for Windows, Version 29.0 (IBM Corp., Armonk, NY, USA).

For LRRFS, only 13 locoregional recurrence events occurred overall (three in Stage I-II and 10 in Stage III). Despite this low event count, stage significantly differentiated LRRFS (log-rank chi-square = 8.248, df = 1, p = 0.004) (Figure 5). Stage III disease was associated with significantly worse LRRFS, although interpretation is limited by the low total number of locoregional recurrence events (n = 13) (Figure 5). By molecular subtype, the LRRFS difference was not statistically significant (log-rank chi-square = 4.147, df = 3, p = 0.246); the HER2-enriched subgroup had zero locoregional recurrence events, further limiting the reliability of subtype-specific LRRFS comparisons. Detailed Kaplan-Meier results are summarised in Table 2.

**Table 2:** Kaplan–Meier and log-rank results for recurrence endpoints. DMFS: distant metastasis-free survival; HER2: HER2-enriched subtype; LumA: Luminal A; LumB: Luminal B; LRRFS: locoregional recurrence-free survival; RFS: recurrence-free survival; TNBC: triple negative breast cancer; NR: not reached/not reliably estimable because of sparse events or insufficient decline in survival probability during follow-up

Endpoint	Comparison	Subgroup	n	Events	Censored	Mean survival, months (95% CI)	p-value	Interpretation
RFS	Stage I–II vs III	Stage I–II	115	26	89	45.39 (43.22–47.57)	<0.001	Stage III worse RFS
RFS	Stage I–II vs III	Stage III	80	38	42	40.17 (36.76–43.58)	<0.001	Stage III worse RFS
RFS	Subtype (4 groups)	Luminal A	112	35	77	44.92 (NR)	0.666	No significant subtype difference
RFS	Subtype (4 groups)	Luminal B	35	10	25	42.47 (NR)	0.666	No significant subtype difference
RFS	Subtype (4 groups)	HER2-enriched	21	8	13	39.06 (NR)	0.666	No significant subtype difference
RFS	Subtype (4 groups)	Triple negative	27	11	16	36.48 (NR)	0.666	No significant subtype difference
DMFS	Stage I–II vs III	Stage I–II	115	19	96	46.83 (44.82–48.85)	0.002	Stage III worse DMFS
DMFS	Stage I–II vs III	Stage III	80	29	51	42.78 (39.39–46.18)	0.002	Stage III worse DMFS
DMFS	Subtype (4 groups)	Luminal A	112	25	87	47.40 (NR)	0.722	No significant subtype difference
DMFS	Subtype (4 groups)	Luminal B	35	9	26	42.96 (NR)	0.722	No significant subtype difference
DMFS	Subtype (4 groups)	HER2-enriched	21	6	15	39.83 (NR)	0.722	No significant subtype difference
DMFS	Subtype (4 groups)	Triple negative	27	8	19	37.89 (NR)	0.722	No significant subtype difference
LRRFS	Stage I–II vs III	Stage I–II	115	3	112	50.95 (NR)	0.004	Stage difference; low events
LRRFS	Stage I–II vs III	Stage III	80	10	70	50.23 (NR)	0.004	Stage difference; low events
LRRFS	Subtype (4 groups)	Luminal A	112	11	101	NR	0.246	No significant difference; sparse events
LRRFS	Subtype (4 groups)	Luminal B	35	1	34	NR	0.246	No significant difference; sparse events
LRRFS	Subtype (4 groups)	HER2-enriched	21	0	21	NR	0.246	No significant difference; sparse events
LRRFS	Subtype (4 groups)	Triple negative	27	1	26	NR	0.246	No significant difference; sparse events

Multivariable Cox regression for RFS

In the primary multivariable Cox model for RFS (covariates: age10, stage group, molecular subtype, surgery type; n = 195), the overall model was statistically significant (omnibus chi-square = 13.997, df = 6, p = 0.030). Stage III disease was independently associated with significantly worse RFS compared with Stage I-II (HR 2.182, 95% CI 1.266-3.761; p = 0.005). Age per 10 years (HR 1.070, 95% CI 0.828-1.381; p = 0.605), molecular subtype (overall p = 0.800), and surgery type (HR 1.338, 95% CI 0.755-2.369; p = 0.318) were not independently associated with RFS.

Sensitivity analyses for RFS

Because the chemotherapy sequence was not randomly assigned and NACT was preferentially used for patients with greater baseline disease burden or biologically aggressive disease, this model should be interpreted as a sensitivity analysis rather than as an estimate of treatment effect. In the treated-only sensitivity model (NACT vs adjuvant chemotherapy; n = 193), the model was statistically significant (omnibus p = 0.002). Stage III remained independently associated with worse RFS (HR 1.957, 95% CI 1.133-3.380; p = 0.016). NACT was also associated with worse RFS compared with adjuvant chemotherapy (HR 2.159, 95% CI 1.209-3.858; p = 0.009), but this association is most plausibly explained by confounding by indication rather than a causal adverse effect of neoadjuvant treatment [8].

In the RT-start-anchored RFS sensitivity model (n = 195), the overall model remained significant (omnibus p = 0.017) and Stage III disease was again independently associated with worse RFS (HR 2.269, 95% CI 1.318-3.906; p = 0.003). Age, molecular subtype, and surgery type were not significantly associated with RFS in this model.

Multivariable Cox regression for DMFS

In the parsimonious DMFS Cox model (age10, stage group, molecular subtype; n = 195; 48 events), the model approached but did not reach omnibus significance (chi-square = 10.595, df = 5, p = 0.060). Although the overall DMFS model did not reach conventional omnibus significance, Stage III disease showed a significant predictor-level association with worse DMFS (HR 2.429, 95% CI 1.337-4.411; p = 0.004), consistent with the primary RFS model. Age (p = 0.825) and molecular subtype (overall p = 0.778) were not significantly associated with DMFS. Full multivariable Cox model results are presented in Table 3. Proportional hazards testing using Schoenfeld residuals showed no evidence of violation for stage group in the primary RFS, treated-only RFS, or DMFS Cox models. However, molecular subtype showed evidence of non-proportionality in the primary RFS and DMFS models, and the global proportional hazards test was significant for the primary RFS and DMFS models. Therefore, Cox results for molecular subtype should be interpreted cautiously.

**Table 3:** Multivariable Cox regression models for recurrence outcomes. BCS: breast-conserving surgery; CI: confidence interval; DMFS: distant metastasis-free survival; HER2: human epidermal growth factor receptor 2; HR: hazard ratio; NACT: neoadjuvant chemotherapy; RFS: recurrence-free survival; RT: radiotherapy. Luminal A, Stage I–II, breast-conserving surgery, and adjuvant chemotherapy were used as reference categories where applicable. Molecular subtype violated the proportional hazards assumption in the primary RFS and DMFS models; therefore, subtype-specific HRs should be interpreted cautiously and should not be used as time-constant estimates of relative risk.

Model	Predictor	HR (95% CI)	p-value	Interpretation
Primary RFS Cox, biopsy-origin	Age per 10 years	1.070 (0.828–1.382)	0.605	Not significant
Primary RFS Cox, biopsy-origin	Stage III vs I–II	2.182 (1.266–3.761)	0.005	Independent predictor of worse RFS
Primary RFS Cox, biopsy-origin	Luminal B vs Luminal A	1.063 (0.524–2.156)	0.865	Not significant
Primary RFS Cox, biopsy-origin	HER2-enriched vs Luminal A	1.189 (0.550–2.569)	0.661	Not significant
Primary RFS Cox, biopsy-origin	Triple negative vs Luminal A	1.410 (0.702–2.832)	0.334	Not significant
Primary RFS Cox, biopsy-origin	Mastectomy vs BCS	1.338 (0.755–2.369)	0.318	Not significant
RFS Cox, treated-only sensitivity	Age per 10 years	1.044 (0.805–1.355)	0.744	Not significant
RFS Cox, treated-only sensitivity	Stage III vs I–II	1.957 (1.133–3.380)	0.016	Stage remains significant
RFS Cox, treated-only sensitivity	Luminal B vs Luminal A	1.116 (0.547–2.277)	0.764	Not significant
RFS Cox, treated-only sensitivity	HER2-enriched vs Luminal A	1.208 (0.556–2.625)	0.633	Not significant
RFS Cox, treated-only sensitivity	Triple negative vs Luminal A	1.321 (0.655–2.664)	0.437	Not significant
RFS Cox, treated-only sensitivity	Mastectomy vs BCS	1.209 (0.676–2.163)	0.522	Not significant
RFS Cox, treated-only sensitivity	NACT vs adjuvant chemotherapy	2.159 (1.209–3.858)	0.009	Higher hazard in NACT group; likely confounding by indication
RFS Cox, RT-start sensitivity	Age per 10 years	1.054 (0.816–1.361)	0.687	Not significant
RFS Cox, RT-start sensitivity	Stage III vs I–II	2.269 (1.318–3.906)	0.003	Stage effect robust to time-origin choice
RFS Cox, RT-start sensitivity	Luminal B vs Luminal A	1.063 (0.524–2.156)	0.865	Not significant
RFS Cox, RT-start sensitivity	HER2-enriched vs Luminal A	1.160 (0.537–2.506)	0.706	Not significant
RFS Cox, RT-start sensitivity	Triple negative vs Luminal A	1.475 (0.736–2.954)	0.273	Not significant
RFS Cox, RT-start sensitivity	Mastectomy vs BCS	1.343 (0.758–2.381)	0.313	Not significant
DMFS Cox, parsimonious	Age per 10 years	0.967 (0.722–1.296)	0.825	Not significant
DMFS Cox, parsimonious	Stage III vs I–II	2.429 (1.337–4.411)	0.004	Independent predictor of worse DMFS
DMFS Cox, parsimonious	Luminal B vs Luminal A	1.369 (0.635–2.953)	0.423	Not significant
DMFS Cox, parsimonious	HER2-enriched vs Luminal A	1.325 (0.541–3.244)	0.538	Not significant
DMFS Cox, parsimonious	Triple negative vs Luminal A	1.360 (0.602–3.076)	0.46	Not significant

In competing-risk sensitivity analysis for DMFS, treating death without prior distant recurrence as a competing event, Stage III disease remained significantly associated with a higher cumulative incidence of distant recurrence compared with Stage I-II disease (sHR 2.392, 95% CI 1.326-4.314; p = 0.004). Age per 10 years was not significantly associated with DMFS (sHR 0.951, 95% CI 0.729-1.242; p = 0.714). Molecular subtype was also not significantly associated with DMFS: Luminal B versus Luminal A (sHR 1.419, 95% CI 0.635-3.170; p = 0.394), HER2-enriched versus Luminal A (sHR 1.326, 95% CI 0.562-3.131; p = 0.519), and triple negative versus Luminal A (sHR 1.352, 95% CI 0.574-3.180; p = 0.490). For LRRFS, an exploratory stage-only Fine-Gray model was performed because only 13 locoregional recurrence events occurred. Stage III disease showed a higher subdistribution hazard of locoregional recurrence compared with Stage I-II disease (sHR 4.998, 95% CI 1.385-18.035; p = 0.014). However, the wide confidence interval indicates substantial imprecision; therefore, this result should be interpreted as exploratory and hypothesis-generating rather than as a stable effect estimate. Full results are presented in Table 4.

**Table 4:** Competing-risk Fine-Gray sensitivity analysis for DMFS and exploratory LRRFS analysis DMFS: distant metastasis-free survival; LRRFS: locoregional recurrence-free survival; sHR: subdistribution hazard ratio; CI: confidence interval. Death without prior endpoint-specific recurrence was treated as a competing event. Luminal A and Stage I-II were the reference categories.

Endpoint	Predictor	sHR	95% CI	p-value
DMFS	Age per 10 years	0.951	0.729–1.242	0.714
DMFS	Stage III vs I–II	2.392	1.326–4.314	0.004
DMFS	Luminal B vs Luminal A	1.419	0.635–3.170	0.394
DMFS	HER2-enriched vs Luminal A	1.326	0.562–3.131	0.519
DMFS	Triple negative vs Luminal A	1.352	0.574–3.180	0.49
LRRFS	Stage III vs I–II	4.998	1.385–18.035	0.014

## Discussion

This study provides real-world recurrence outcome data from a South Asian LMIC cancer centre, where institutional outcomes after adjuvant breast radiotherapy remain underreported. The estimated three-year RFS was 78.1% for Stage I-II disease, and 60.0% for Stage III disease, and the estimated three-year DMFS was 85.0% and 70.7%, respectively. Across unadjusted Kaplan-Meier analyses and adjusted multivariable Cox models, tumour stage consistently emerged as the most robust predictor of recurrence outcomes. Stage also appeared to differentiate LRRFS; however, this finding should be considered exploratory because only 13 locoregional recurrence events occurred, and the Fine-Grey estimate was imprecise, with a wide CI. These findings are broadly consistent with the established relationship between stage and recurrence risk in breast cancer and with prior reports showing that higher-risk disease is associated with poorer outcomes after radiotherapy [9].

The consistent association between Stage III disease and poorer RFS and DMFS is clinically plausible because a higher stage reflects greater tumour burden, nodal involvement, and a higher baseline risk of systemic relapse despite curative-intent multimodality treatment. Importantly, the stage effect persisted after multivariable adjustment and in the radiotherapy-start-anchored sensitivity analysis, and proportional hazards testing confirmed that the assumption was not violated for stage group, supporting the robustness of this finding and reducing the likelihood that it was an artifact of time-origin selection. Although the DMFS parsimonious Cox model did not reach omnibus significance (p = 0.060), Stage III disease showed a significant predictor-level association with worse DMFS (HR 2.429, 95% CI 1.337-4.411; p = 0.004), a finding that was further corroborated by the competing-risk Fine-Gray analysis.

Although the association between higher stage and poorer recurrence outcomes is well established, this study contributes locally relevant outcome benchmarks from a real-world Pakistani cohort. Published three-year recurrence estimates from comparable LMIC settings are sparse, and stage distribution, referral timing, treatment sequencing, systemic therapy completion, and follow-up patterns in such populations may diverge substantially from those observed in trial cohorts or high-income registry datasets. The present estimates may therefore serve as a reference point for clinical risk stratification and follow-up planning at similar institutions. Available regional literature has more commonly reported overall or stage-specific survival in broad breast cancer populations rather than recurrence-specific outcomes after adjuvant radiotherapy. For example, Maajani et al. systematically reviewed one-, three-, five-, and 10-year breast cancer survival across countries in the Eastern Mediterranean Region, while Kumar et al. reported presentation, treatment patterns, and survival outcomes from Pakistan; however, neither study directly addresses three-year RFS, DMFS, and LRRFS in a uniformly adjuvant-radiotherapy-treated Stage I-III cohort [10,11].

The absence of a statistically significant association between molecular subtype and recurrence endpoints should be interpreted cautiously and is more plausibly attributable to statistical limitations than to true biological equivalence. Subtype-specific subgroups were small (HER2-enriched n = 21, triple-negative n = 27), event counts were low, and trastuzumab and endocrine therapy completion data were incomplete, all of which would be expected to attenuate subtype-specific associations. Prior studies in larger patient series have demonstrated meaningful subtype differences in recurrence timing, locoregional relapse risk, and metastatic behaviour, with triple-negative and HER2-enriched tumours showing earlier and more aggressive relapse trajectories and Luminal A tumours generally associated with more favourable outcomes [6,12-16]. Ribelles et al. showed that recurrence hazard varies over time according to intrinsic subtype and proliferation index [13]. Voduc et al. reported differences in local and regional relapse risk across biologic subtypes [14]. Dent et al. described the characteristic early-recurrence pattern of triple-negative breast cancer [15], while Kennecke et al. demonstrated subtype-specific differences in metastatic behaviour [16]. The direction of the observed mean RFS differences in our cohort, numerically shorter in HER2-enriched and triple-negative subgroups, is consistent with these established patterns, even though statistical significance was not reached. Because molecular subtype showed evidence of non-proportional hazards in the primary RFS and DMFS Cox models, the subtype-specific hazard ratios in Table 3 should not be interpreted as stable point estimates of relative risk over time. They are reported as adjusted exploratory summaries, while the Kaplan-Meier patterns, event counts, and confidence intervals should guide interpretation.

The association between NACT and worse RFS in the treated-only sensitivity model should not be interpreted as evidence of harm from neoadjuvant chemotherapy. NACT is preferentially used in patients with greater tumour burden, nodal involvement, locally advanced disease, or biologically aggressive tumours, introducing confounding by indication that cannot be fully addressed in this retrospective dataset. Propensity-score adjustment was not performed because the study was not designed to estimate the causal effect of chemotherapy sequence, and the available event count and covariate details were limited. This result should therefore be interpreted as a hypothesis-generating reflection of differential baseline risk rather than as an indication of treatment-related harm.

A methodological strength of this study is the use of complementary time-to-event analyses with consistent endpoint definitions, together with competing-risk sensitivity analyses and an RT-start-anchored sensitivity model for the primary endpoint. The study contributes real-world recurrence estimates from a South Asian cancer centre, a setting that remains under-represented in the peer-reviewed breast radiotherapy outcomes literature. More broadly, the clinical relevance of identifying residual recurrence risk after adjuvant radiotherapy is well supported by prior evidence. In the Early Breast Cancer Trialists' Collaborative Group (EBCTCG) meta-analyses, radiotherapy after breast-conserving surgery and after mastectomy substantially reduced both recurrence and breast cancer mortality, underscoring the importance of local therapy in early breast cancer management [3,17]. Against that background, our findings suggest that even within a uniformly radiotherapy-treated cohort, stage remains a dominant discriminator of recurrence risk, a finding particularly relevant in real-world practice, where treatment completion, referral patterns, and follow-up intensity may vary more than in trial populations. Future multicentre studies with larger subtype-specific samples, standardised data capture for anti-HER2 therapy and endocrine treatment completion, and longer follow-up are needed to more robustly characterise subtype-specific recurrence patterns in LMIC settings.

Limitations

This study has several limitations. First, its retrospective single-centre design introduces inherent selection bias and residual confounding that cannot be fully eliminated without randomisation or propensity-score adjustment, and no a priori sample size calculation was performed, given the consecutive cohort design.

Second, statistical power was limited by the moderate overall cohort size (n = 195) and small subtype-specific subgroups, particularly HER2-enriched disease (n = 21) and triple-negative disease (n = 27). Low event counts, particularly for LRRFS (n = 13), precluded reliable multivariable modelling for that endpoint and reduced power to detect clinically meaningful subtype differences; consequently, the LRRFS Fine-Gray model was limited to stage group only and should be interpreted cautiously. Ki-67 was available only as a binary institutional category rather than as a continuous value, meaning Luminal A/B classification relied on a simplified surrogate that may have introduced subtype misclassification and attenuated subtype-specific associations.

Third, follow-up duration was insufficient to capture long-term recurrence risk, particularly in the predominantly luminal cohort. The present findings should therefore be interpreted as early three-year outcomes rather than definitive long-term estimates. Incomplete trastuzumab and endocrine therapy completion data also prevented adjustment for systemic treatment intensity, an important determinant of subtype-specific recurrence risk.

Finally, proportional hazards testing indicated possible non-proportionality for molecular subtype, consistent with known differences in recurrence timing across breast cancer subtypes, and subtype-specific hazard ratios should therefore be interpreted with caution. Only four patients had involved surgical margins, none of whom developed locoregional recurrence, so margin status was not included in multivariable models. The biopsy date was used as the primary time origin, although sensitivity testing from radiotherapy start supported the robustness of the main stage-related findings.

## Conclusions

In this single-centre 2022 cohort of Stage I-III breast cancer patients treated with adjuvant radiotherapy, tumour stage was the most consistent and robust predictor of recurrence outcomes. Stage III disease was associated with significantly worse RFS and DMFS in both unadjusted and adjusted analyses and with worse LRRFS on event-limited Kaplan-Meier analysis. Molecular subtype was not independently associated with recurrence endpoints in this cohort, most likely reflecting limited subgroup sizes, low event counts, and incomplete systemic treatment data rather than true biological equivalence across subtypes. These results support the use of stage as the primary risk stratification tool in routine practice and underscore the importance of developing larger multicentre datasets with complete systemic treatment records to clarify subtype-specific recurrence risk in South Asian and LMIC breast cancer populations.

## References

[1] (2026). Breast cancer. https://www.who.int/news-room/fact-sheets/detail/breast-cancer.

[2] GBD 2023 Breast Cancer Collaborators (2026). Global, regional, and national burden of breast cancer among females, 1990-2023, with forecasts to 2050: a systematic analysis for the Global Burden of Disease Study 2023. Lancet Oncol.

[3] Darby S, McGale P, Correa C (2011). Effect of radiotherapy after breast-conserving surgery on 10-year recurrence and 15-year breast cancer death: meta-analysis of individual patient data for 10,801 women in 17 randomised trials. Lancet.

[4] Whelan TJ, Olivotto IA, Parulekar WR (2015). Regional nodal irradiation in early-stage breast cancer. N Engl J Med.

[5] Senkus E, Kyriakides S, Ohno S (2015). Primary breast cancer: ESMO Clinical Practice Guidelines for diagnosis, treatment and follow-up. Ann Oncol.

[6] Huber KE, Carey LA, Wazer DE (2009). Breast cancer molecular subtypes in patients with locally advanced disease: impact on prognosis, patterns of recurrence, and response to therapy. Semin Radiat Oncol.

[7] von Elm E, Altman DG, Egger M, Pocock SJ, Gøtzsche PC, Vandenbroucke JP (2008). The Strengthening the Reporting of Observational Studies in Epidemiology (STROBE) statement: guidelines for reporting observational studies. J Clin Epidemiol.

[8] Xu Y, Liu B, Chen C (2025). Impact of neoadjuvant and adjuvant chemotherapy on breast cancer prognosis in a propensity score matched population. Sci Rep.

[9] Kim KJ, Huh SJ, Yang JH (2005). Treatment results and prognostic factors of early breast cancer treated with a breast conserving operation and radiotherapy. Jpn J Clin Oncol.

[10] Maajani K, Khodadost M, Fattahi A, Pirouzi A (2020). Survival rates of patients with breast cancer in countries in the Eastern Mediterranean Region: a systematic review and meta-analysis. East Mediterr Health J.

[11] Kumar S, Shaikh AJ, Rashid YA (2016). Presenting features, treatment patterns and outcomes of patients with breast cancer in Pakistan: experience at a university hospital. Indian J Cancer.

[12] Pogoda K, Niwińska A, Murawska M, Pieńkowski T (2013). Analysis of pattern, time and risk factors influencing recurrence in triple-negative breast cancer patients. Med Oncol.

[13] Ribelles N, Perez-Villa L, Jerez JM (2013). Pattern of recurrence of early breast cancer is different according to intrinsic subtype and proliferation index. Breast Cancer Res.

[14] Voduc KD, Cheang MC, Tyldesley S, Gelmon K, Nielsen TO, Kennecke H (2010). Breast cancer subtypes and the risk of local and regional relapse. J Clin Oncol.

[15] Dent R, Trudeau M, Pritchard KI (2007). Triple-negative breast cancer: clinical features and patterns of recurrence. Clin Cancer Res.

[16] Kennecke H, Yerushalmi R, Woods R (2010). Metastatic behavior of breast cancer subtypes. J Clin Oncol.

[17] McGale P, Taylor C, Correa C (2014). Effect of radiotherapy after mastectomy and axillary surgery on 10-year recurrence and 20-year breast cancer mortality: meta-analysis of individual patient data for 8135 women in 22 randomised trials. Lancet.

